# Nutritional Risk Screening in Hospitalized Adults Using the Malnutrition Universal Screening Tool at a Tertiary Care Hospital in South India

**DOI:** 10.7759/cureus.24681

**Published:** 2022-05-02

**Authors:** Arankesh Mahadevan, Hariharan Eswaran, Meenakshi Sundari

**Affiliations:** 1 Internal Medicine, SRM (Sri Ramaswamy Memorial) Medical College Hospital & Research Centre, Kattankulathur, IND

**Keywords:** body mass index, hospitalized patients, nutritional risk screening, socioeconomic status, malnutrition universal screening tool

## Abstract

Background and objectives

Malnutrition is still widely prevalent in India. Various nutritional screening tools have been developed to screen for nutritional risk status but no one tool is considered the best. The Malnutrition Universal Screening Tool (MUST) is accepted by the European Society for Clinical Nutrition and Metabolism and validated for use in hospitalized adults. Hence, it was used in this study to estimate the prevalence of malnutrition in hospitalized adults and its association with socioeconomic inequality.

Methods

A sample of randomly selected 358 ambulatory hospitalized patients above 18 years of age was used in the study. Data pertaining to demography, socioeconomic status, medical history, and MUST were collected using a structured questionnaire. The height and weight of the patients were measured, and their BMI was determined. The patients were classified into five socioeconomic classes and their MUST scores were determined.

Results

Statistically significant (P < 0.05) increasing trend was observed in the height, weight, and BMI of patients with increasing socioeconomic status. Diabetes mellitus (39%) followed by hypertension (30%) were the predominant comorbid conditions. According to MUST, the overall prevalence of medium and high risk of malnutrition was 11% and 24%, respectively, and the socioeconomic class that was most impacted was Class 4 (1,130-2,259 INR per capita monthly income).

Interpretation and conclusions

Socioeconomic status influences the prevalence of malnutrition, comorbid conditions, and the anthropometric measurements of admitted patients. The prevalence of nutritional risk status irrespective of sex was found to be 34.91% (24.3% in males and 10.61% in women) in the study.

## Introduction

Malnutrition is a condition in which the body’s nutritional requirements are unmet due to underconsumption or impaired absorption [1]. According to the Global Nutritional Report 2020, India is among 88 countries that are expected to miss all the global nutritional targets by 2025 set by the World Health Assembly [2]. The magnitude of malnutrition in India is underreported and is further complicated by a lack of consensus on diagnostic criteria for application in clinical settings. A study by the NCD Risk Factor Collaboration (NCD-RisC) reports that although the prevalence of moderate and severe underweight has decreased worldwide from 9·2% in 1975 to 8·4% in 2016 in girls and from 14·8% in 1975 to 12·4% in 2016 in boys, the prevalence of moderate and severe underweight was still highest in India, at 22·7% among girls and 30·7% among boys in the age group of 5 to 19 years [3]. Intervention to improve the current situation of poor nutritional status in the country requires a quick and accurate diagnosis of malnutrition. The presence of various nutritional screening tools for the diagnosis of at nutritional risk status, both validated and not validated, produce varying results. There is no consensus on a single ‘best’ tool and the use of different tools in different studies hinders the ability to make conclusions [4]. The European Society for Clinical Nutrition and Metabolism (ESPEN) suggests the use of the Malnutrition Universal Screening Tool (MUST), which is a validated screening tool to identify adults who are malnourished or at risk of malnutrition (undernutrition) [5,6].

There exists significant socioeconomic inequality in malnutrition; studies have shown an inverse relationship between malnutrition and the economic development of local districts of India. A study analyzing India’s National Health Family Survey (2005-2006) concluded that higher wealth is associated with a lower likelihood of being underweight across all sub-populations [7,8]. Various scales have been developed in India to assess the socioeconomic status of populations, both urban and rural. The BG Prasad scale is used to estimate the socioeconomic status of individuals in both urban and rural settings using just per-capita monthly income and is revised yearly based on the consumer price index (CPI) updated by the Government of India [9,10]. The presence of comorbid conditions, viz., diabetes mellitus, hypertension, chronic kidney disease, etc., significantly worsens malnutrition, the exact reasons being multifactorial. The situation can be further aggravated by hospitalization as patients often receive less than optimal nutrition during their stay. Previous studies have associated a higher prevalence of malnutrition in patients with a higher Charlson Comorbidity Index [11]. Hence, this study was designed to evaluate the prevalence of malnutrition and its association with socioeconomic inequality in the presence or absence of comorbid conditions using a validated nutritional screening tool at a tertiary care hospital in Chengalpet district, Tamil Nadu, India.

## Materials and methods

A cross-sectional study was undertaken with patients admitted to SRM Medical College Hospital and Research Centre, Kattankulathur, Tamil Nadu, India. The study was approved by the institutional ethics committee of the SRM Medical College Hospital & Research Centre, Kattankulathur, Tamil Nadu, India (approval number: 2901/IEC/2021). Voluntary written informed consent was taken from all the patients admitted for the study. 

Of the total patients admitted to the hospital between August 2021 to December 2021, 358 patients were randomly selected for the study. This sample size of 358 was determined using the sample size formula usually used for qualitative variables in cross-sectional studies or cross-sectional surveys for an expected prevalence of 37.1% [12,13]. Sample Size (n) = $(p(1-p) z^2)/E^2$.

The inclusion criteria adopted for the selection of patients for this study were all patients above 18 years of age who were admitted and from whom consent was obtained. Patients who were not ambulatory, were pregnant, and/or who did not provide consent for data collection were excluded from the study. 

A pilot-tested structured questionnaire was used to document the data from the selected patients by the research team at the patient’s bedside. The questionnaire included demographic data (age and sex), socioeconomic status information (family income, number of family members), medical condition of the patient (primary diagnosis and comorbid conditions), and data about MUST that included two important criteria: history of unplanned weight loss in the past six months, which was classified as > 10%, 10 to 5%, < 5% body weight, and acute illness of patient or patient having no nutritional intake for > 5 days. 

In addition, anthropometric data from the patients were collected as per CDC, 2020. Height was measured using a two-meter stadiometer and measurements made up to the nearest 0.1cm, with patients standing barefoot, back straight, ankle, buttocks, shoulders, occiput touching stadiometer, and Frankfort horizontal plane parallel to floor and height was expressed in meters (m). Weight was measured using a standard weighing scale to the nearest 0.01kg with patients wearing as minimal clothing as possible and weight was expressed in kilograms (kg). From the measurements of height and weight, BMI (kg/m2) was calculated [14].

From the data, patients were divided into socioeconomic groups using the updated 2020 BG Prasad Socioeconomic Status Classification as it is applicable for both rural and urban populations in India [9]. MUST score was calculated and patients were classified as low risk (score = 0), medium risk (score = 1), and high risk (score >= 2). Medium risk and high risk patients were categorized as at nutritional risk [6].

The data were analyzed with analysis of variance (ANOVA) and linear regression analysis using IBM SPSS Statistics for Windows, Version 28.0 (Released 2021; IBM Corp., Armonk, New York, United States). The critical difference between the groups was analyzed using Duncan’s multiple range tests and was presented in tables indicated by suitable alphabetical superscripts.

## Results

The study involved a total of 358 patients, of which 238 (66.5%) were male and 120 (33.5%) were females. The details of the selected patients for the study are presented in Table 1. 

**Table 1:** Details of the patients selected for the study (Mean ± SE)

		Male	Female	Total
1.	Number of patients	238 (66%)	120 (34%)	358
2.	Age (Years)	51 ± 15	45 ± 12	49 ± 14
3.	Socioeconomic class (income per capita/month)			
	Class 1 ( > 7,533 INR)	35 (10%)	3 (1%)	33 (11%)
	Class 2 (3,766-7,532 INR)	68 (19%)	27 (8%)	95 (27%)
	Class 3 (2,260-3,765 INR)	63 (18%)	54 (15%)	117 (33%)
	Class 4 (1,130-2,259 INR)	51 (14%)	23 (7%)	74 (21%)
	Class 5 ( < 1,130 INR)	21 (6%)	13 (3%)	34 (9%)
4.	Department of admission			
	Medical departments	135 (38%)	90 (25%)	225 (63%)
	Surgical departments	103 (29%)	30 (8%)	133 (37%)
5.	Co-morbidities			
	Diabetes mellitus	90 (25%)	49 (14%)	139 (39%)
	Hypertension	79 (22%)	29 (8%)	108 (30%)
	Cardiovascular diseases	37 (10%)	8 (3%)	45 (13%)
	Thyroid diseases	10 (3%)	25 (7%)	35 (10%)
6.	Nutritional characteristics			
	Height (m)	1.637 ± 0.070	1.526 ± 0.068	1.600 ± 0.087
	Weight (Kg)	66.367 ± 14.716	56.881 ± 12.769	63.188 ± 14.772
	Body mass index (Kg/m^2^)	24.395 ± 5.327	24.455± 5.443	24.415 ± 5.359

**Figure 1:**
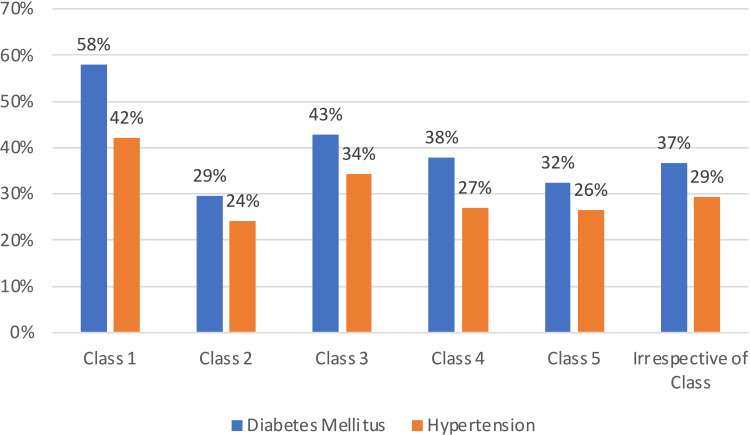
Prevalence of diabetes and hypertension in different socioeconomic classes

The predominant socioeconomic class amongst males was Class 2 (19%), in females it was Class 3 (15%), and irrespective of sex it was Class 3 (33%). The majority of patients were admitted to the medical departments (63%). The predominant comorbidity observed in males (25%), females (14%), and irrespective of sex (39%) was diabetes mellitus followed by hypertension, which was observed in 30% of the total patients. It was observed that men on average were taller (1.637 vs. 1.526 m) and heavier (66.367 vs. 56.881 kg) than women, but their BMI (24.395 vs 24.455 kg/m2) was significantly lower (p < 0.01) than that of women. The prevalence of comorbidities such as diabetes and hypertension in different socioeconomic classes is depicted in Figure 1.

The overall prevalence of diabetes and hypertension was found to be 39% and 30% respectively. The highest prevalence of diabetes and hypertension was observed in Class 1. The anthropometric measurements concerning socioeconomic classes in the patients selected for the study are presented in Table 2.

**Table 2:** Anthropometric measurements concerning socioeconomic classes in the patients selected for the study (Mean ± SE) ** Significant at 1% level The superscripts are mentioned based on the Duncan homogenous subsets Height: a - Class 4 and Class 5 are homogenous sub-sets and they significantly differ from other classes b - Class 2, Class 3, and Class 4 are homogenous sub-sets and they significantly differ from other classes c - Class 5 significantly differs from other classes Weight: d - Class 4 and Class 5 are homogenous sub-sets and they significantly differ from other classes e - Class 2 and Class 3 are homogenous sub-sets and they significantly differ from other classes f - Class 5 significantly differs from other classes BMI: g - Class 4 and Class 5 are homogenous sub-sets and they significantly differ from other classes h - Class 2 and Class 5 are homogenous sub-sets and they significantly differ from other classes i - Class 1, Class 2, and Class 3 are homogenous sub-sets and they significantly differ from other classes

Socioeconomic Class	N	Height (m)	Weight (Kg)	BMI (Kg/m^2^)
Class 1 ( > 7,533 INR)	38	1.664^c^ ± 0.086	73.834^f^ ± 15.942	26.476^i^ ± 4.636
Class 2 (3,766-7,532 INR)	95	1.605^b^ ± 0.084	64.964^e^ ± 15.318	24.812^hi^ ± 5.924
Class 3 (2,260-3,765 INR)	117	1.596^b^ ± 0.085	64.988^e^ ± 12.751	25.548^i^ ± 4.949
Class 4 (1,130-2,259 INR)	95	1.584^ab^ ± 0.085	55.804^d^ ± 13.498	21.679^g^ ± 5.063
Class 5 ( < 1,130 INR)	34	1.561^a^ ± 0.071	56.202^d^ ± 10.453	23.064^gh^ ± 3.906
Irrespective of socioeconomic class	358	1.600^**^ ± 0.087	63.188^**^ ± 14.772	24.415^**^ ± 5.359
F value		8.119	14.018	8.933
P value		< 0.01	< 0.01	< 0.01

Significant variation (P < 0.05) in height, weight, and BMI was observed between socioeconomic classes. Significantly (P < 0.05) shortest (1.561 ± 0.071 m) and tallest (1.664 ± 0.086 m) patients belonged to Class 1 and Class 5, respectively. Significantly lowest (P < 0.05) body weight was observed in Class 4 (55.804 ± 13.498 kg) and Class 5 (56.202 ± 10.453 kg), and significantly highest (P < 0.05) body weight was observed in Class 1 (73.834 ± 15.942 kg). The significantly lowest (P < 0.05) BMI was observed in Class 4 (21.679 ± 5.063 kg/m2), and the significantly highest (P < 0.05) BMI in Class 3 (25.548 ± 4.949 kg/m2) and Class 1 (26.476 ± 4.636 kg/m2). Class 4 and Class 5 had the optimal BMI as per WHO. The distribution of patients in MUST categories as per gender and socioeconomic class is presented in Table 3.

**Table 3:** Distribution of patients in Malnutrition Universal Screening Tool (MUST) categories as per gender and socioeconomic class

		Socioeconomic Class					
	Risk of Malnutrition	Class 1	Class 2	Class 3	Class 4	Class 5	Irrespective of Class
Male	Low (Score=0)	28 (8%)	41 (11%)	47 (13%)	23 (6%)	13 (3%)	151 (42%)
	Medium (Score=1)	0 (0%)	11 (3%)	2 (1%)	10 (3%)	1 (0%)	24 (7%)
	High (Score>=2)	7 (2%)	16 (4%)	14 (4%)	18 (5%)	8 (2%)	63 (18%)
Female	Low (Score=0)	3 (1%)	17 (5%)	44 (12%)	8 (2%)	10 (3%)	82 (23%)
	Medium (Score=1)	0 (0%)	5 (1%)	4 (1%)	7 (2%)	0 (0%)	16 (4%)
	High (Score>=2)	0 (0%)	5 (1%)	6 (2%)	8 (2%)	3 (1%)	22 (6%)
Irrespective of Sex							
	Low (Score=0)	31 (9%)	58 (16%)	91 (25%)	31 (9%)	22 (6%)	233 (65%)
	Medium (Score=1)	0 (0%)	16 (4%)	6 (2%)	17 (5%)	1 (0%)	40 (11%)
	High (Score>=2)	7 (2%)	21 (6%)	20 (6%)	26 (7%)	11 (3%)	85 (24%)

The number of men at medium and high risk of malnutrition was 24 (7%) and 63 (18%), respectively. This was more than that of women, which was 16 (4%) and 22 (6%), respectively. The socioeconomic class with most patients at medium and high risk of malnutrition was Class 4. Irrespective of socioeconomic class and sex, the number of patients at medium and high risk of malnutrition was 40 (11%) and 85 (24%), respectively. The overall prevalence of patients at nutritional risk was found to be 34.91%.

## Discussion

Based on the Global Hunger Index report 2021, India ranked 101 among the 116 countries indexed, which indicates the high prevalence of under-nutrition and hunger-related problems in the country [15]. Nutritional risk status assessment is currently undertaken using various nutritional screening tools (NST). The MUST shows good sensitivity and specificity compared to other NSTs for hospitalized patients (Nutritional Risk Screening Tool 2002, Short Nutritional Assessment Questionnaire, etc.,) [4]. Cederholm et al. 2017 also suggested the use of the MUST and the Nutritional Risk Screening Tool 2002 (NRS-2002) over other available screening tools to identify patients at nutritional risk [5]. However, a limited number of studies have estimated nutritional risk using the MUST in the Indian population [16,17] and, hence, this tool was chosen for the current study. 

In day-to-day life, socioeconomic status is an important factor that affects the health condition of an individual, his/her family, social security, and family health status [9]. Hence, in this study, the patients were grouped under five socioeconomic classes as per the modified BG Prasad scale 2020. It was observed that 33% of the patients studied belonged to Class 3 and had a per capita monthly income of 2,260-3,765 INR in contrast to the average per capita income of individuals in urban areas of Tamil Nadu, which is 5,520 INR placing them in Class 2 of modified the BG Prasad scale 2020 [18]. As the patients of the study were from semi-urban/rural areas of Tamil Nadu, their per capita monthly income could have been lower. Socioeconomic Class 5 (< 1,130 INR) had the least number of patients at nutritional risk; this could be attributed to a higher prevalence of labour-intensive occupations incurring a higher daily energy expenditure.

Incidence of diabetes mellitus in the study population was found to be 39%, which concurs with the report by Arun Nanditha et al. in which the prevalence of diabetes was found to increase from 18.6% to 21.9% in the city, 16.4% to 20.3% in the town, and 9.2% to 13.4% in the peri-urban village [19]. Concurring with this study that hypertension was prevalent in 30% of the individuals studied, Oommen et al. also reported that 29% and 31% of individuals living in urban and rural areas, respectively, of Vellore district, Tamil Nadu, India, had hypertension [20]. Furthermore, it was evident that the prevalence of comorbidities (diabetes and hypertension) was higher in socioeconomic Class one (income per capita per month > 7,533 INR). This observation is similar to that reported by Daniel et al., who in their study had also concluded that higher socioeconomic status predisposed to population burden of diabetes and hypertension [21]. 

This study further reinstates the fact that socioeconomic status plays a primary role in determining the height, weight, and BMI of individuals. Young et al. too reported that factors associated with a lower prevalence of underweight among women included higher socioeconomic status, urban residence, and improved diet diversity and latrine use. Higher education levels, decision-making, and ownership of money were also associated with a lower prevalence of underweight [22]. Socioeconomic status influences BMI through multiple factors, which include purchasing power, differences in food consumption patterns, and cultural norms on body size ideals. The BMI of patients studied, regardless of sex, was not optimum to that recommended by WHO; WHO suggests a BMI of 23-27·5 kg/m2 as increased risk [23].

Patients belonging to the medium and high risk of malnutrition categories according to the MUST were considered malnourished in this study. The prevalence of malnutrition, irrespective of sex, was found to be 34.91%. Other studies performed on hospitalized adults in India [24,25] using different sets of NSTs have provided different estimates of prevalence, adding to the fact that no single NST is adequate to define malnutrition on a global scale in all patient environments. The Global Leadership Initiative on Malnutrition (GLIM) criteria for the definition of malnutrition attempt to address this issue by adopting a two-step approach for the diagnosis of malnutrition [26]. The first step is to identify nutritional risk status using standardized NSTs like MUST. The second step requires the identification of one phenotypic and one etiological criterion [26]. Application of the GLIM criteria on the ground level is hard due to the complexity of the process and difficulty in obtaining some of the phenotypical and etiological criteria.

The limitation of the current study is that it covered only a population of 358 hospitalized adults at a single tertiary care hospital located in a periurban area. The study needs further validation with a larger population covering multiple hospitals, both rural and urban. 

## Conclusions

The socioeconomic status of the individuals plays a primary role in their height, weight, and BMI. Socioeconomic classes with lower income have a higher prevalence of nutritional risk status, and Class 5 (Income < 1,130 INR per capita per month) had the lowest anthropometric measurements. The prevalence of nutritional risk, irrespective of sex, was found to be 34.91%. There was a high incidence of diabetes mellitus (39%) and hypertension (30%) in the patients admitted to the study, with higher-income groups showing a higher prevalence of co-morbidities.
